# Putative native South Amerindian origin of head lice clade F: evidence from head lice nits infesting human shrunken heads

**DOI:** 10.1038/s41598-022-08176-2

**Published:** 2022-03-12

**Authors:** Nadia Amanzougaghene, Philippe Charlier, Florence Fenollar, Didier Raoult, Oleg Mediannikov

**Affiliations:** 1 IHU-Méditerranée Infection, Marseille, France; 2 Aix Marseille Université, IRD, AP-HM, MEPHI, Marseille, France; 3 Laboratory «Anthropology, Archaeology, Biology» (LAAB), Université Paris-Saclay, UVSQ, 78180 Montigny-le-Bretonneux, France; 4 Musée du quai Branly – Jacques Chirac, 222 rue de l’Université, 75007 Paris, France; 5 Aix Marseille Université, IRD, AP-HM, SSA, VITROME, Marseille, France

**Keywords:** Molecular evolution, Phylogenetics

## Abstract

The head louse, *Pediculus humanus capitis*, is a strictly obligate human ectoparasite with a long history of association with humans. Here, 17 ancient head lice nits were recovered from six shrunken human heads (known as *tsantsas*) of individuals from the Shuar/Jivaro tribe, a native Amazonian population from Ecuador, South America. *Cytochrome b* DNA analysis revealed the presence of three known mitochondrial clades. Clade A was the most frequent (52.94%), followed by F (35.29%), and B (11.76%). Eleven haplotypes were found in 17 samples, and nine of the haplotypes were novel, indicating an unusually high genetic diversity. In conclusion, we confirmed the presence of clades A, B and F in South Amerindian population. Moreover, the description of clade F, together with its previous reports in another Amerindian population from French Guiana, strongly support the hypothesis of a native South American origin for this clade, and probably derived from clade B which was carried to America by an ancestral Eurasian Beringian population. Further support to our conclusion and new insights might come from the analysis of a larger collection of modern and ancient native American lice.

## Introduction

Head lice, *Pediculus humanus capitis*, and body lice, *Pediculus h. humanus*, are two ecotypes of the same species that feed exclusively on human blood^1,2^. Head lice live among human hairs and feed on blood from the scalp. Head lice are common and can be found worldwide, mainly in school-aged children^1,3^. Body lice live in clothing, are associated with poor socio-economic conditions, and are less prevalent than head lice^1,4^. The body louse is the main vector of at least three serious human diseases which are responsible for the deaths of several million people: *Rickettsia prowazekii* (the causative agent of epidemic typhus), *Bartonella quintana* (trench fever), and *Borrelia recurrentis* (relapsing fever)^1,5^. It is also strongly suspected to transmit *Yersinia pestis*, the causative agent of plague^6–8^. Moreover, several recent studies have also implicated head lice as an additional potential vector of pathogens, although its vectorial capacity is lower than that of body lice^9–15^. For instance, head lice have been shown to be able to acquire, maintain and transmit *R. prowazekii* and *B. quintana* under experimental conditions^13,16,17^. Moreover, DNA of several pathogenic bacteria has been found in head lice, such as, *B. quintana*, *B. recurrentis*, *Y. pestis*, *C. burnetii*, *R. aeschlimannii* and *Acinetobacter* spp.^9–11,15,18^.

Phylogenetically, head lice are classified into six divergent mitochondrial clades (A, B, C, D, E, and F), while body lice belong only to clades A and D^1,19,20^. Clade A is distributed worldwide; clade D has only been reported in the Democratic Republic of Congo (Congo- Kinshasa**)**, the Republic of Congo (Congo-Brazzaville), Zimbabwe and Ethiopia^9,19,21^. Clade B is observed in America, Western Europe, Australia, north Algeria, South Africa, Saudi Arabia, and Iran^20,22–25^. Clade F appears to be specific to South America, as it has not been reported elsewhere^19^. Clade C is, to date, limited to Africa and Asia^9,20,26,27^. Lastly, clade E has mainly been found in West African countries^10,19,28^.

*Pediculus* lice are among oldest human parasites and have a long history of association with humans. They accompanied early *Homo* groups as they migrated out of Africa^1,2,29^. As such they represent good markers for tracking human history^2^. Indeed, phylogenetic analyses of *Pediculus* lice have confirmed some events in the human evolution, such as the estimation date of *H. sapiens* when began wearing clothing, by estimating the age of the body louse (approximately 170,000 years ago), which would first emerged only after the start of clothing use by humans, since the female body louse lays eggs exclusively on the host’s clothing^30,31^. Moreover, lice population show the signs of a recent demographic expansion that occurred roughly 100,000 years ago, coinciding with the spread of *H. sapiens* out of Africa^2,22^. Thus, allowing the use of lice to resolve some of the issues related to our understanding of human migration, such as the timing and trajectories of New Word colonization^1,22^. In recent years, several fossil records of lice and nits from different archaeological sites have been expanded^32^. The earliest ancient specimen of head lice nits, dating back 10,000 years, was found in Brazil, South America^29^. Analysis of ancient DNA is a useful tool for elucidating past events in human migration and evolutionary history^1,32^. However, few studies of ancient DNA have been carried out on these archaeological findings. Thus, based on molecular analyses of louse nits from Israel dating from the Chalcolithic and early Islamic period, Drali et al*.* showed that these specimens may have belonged to people originating from West Africa, as they belonged to the louse mitochondrial clade E (initially classified as sub-clade C) specific to that region^32^. Molecular studies have also been carried out in ancient head lice from Peruvian mummies, showing that clades A and B had a pre-Columbian presence on the American continent^33,34^, suggesting an American origin for clade B^33^. However, this hypothesis has been challenged by its recent discovery among the remains of head lice found in Israel, dating back about 2,000 years^35^. In that study, the authors strongly argued in favour of an Asian origin of clade B, that resulted probably from a recent host switch from Neanderthals or Denisovans to modern humans during periods of overlap, which was followed by its introduction to the New World with the first people who set up there^35^. Moreover, its nearest neighbor clade F was suggested to be Native American origin, since this clade was found to be the most common lineage in the Amazonian lice and have never been found in Asia or any other region reputed to have led to the peopling of the Americas^19^. Therefore, a more detailed analysis of genetic diversity in *P. humanus* infesting native American population will provide insights into the evolutionary pattern of lice clades, their origin and will clarify additional events of human colonization of the Americas.

In this study, we obtained and analysed the genetic diversity of ancient head lice nits collected from shrunken heads of individuals from the Shuar/Jivaro tribe, a native Amazonian population from Ecuador.

## Results

**Figure 1:**
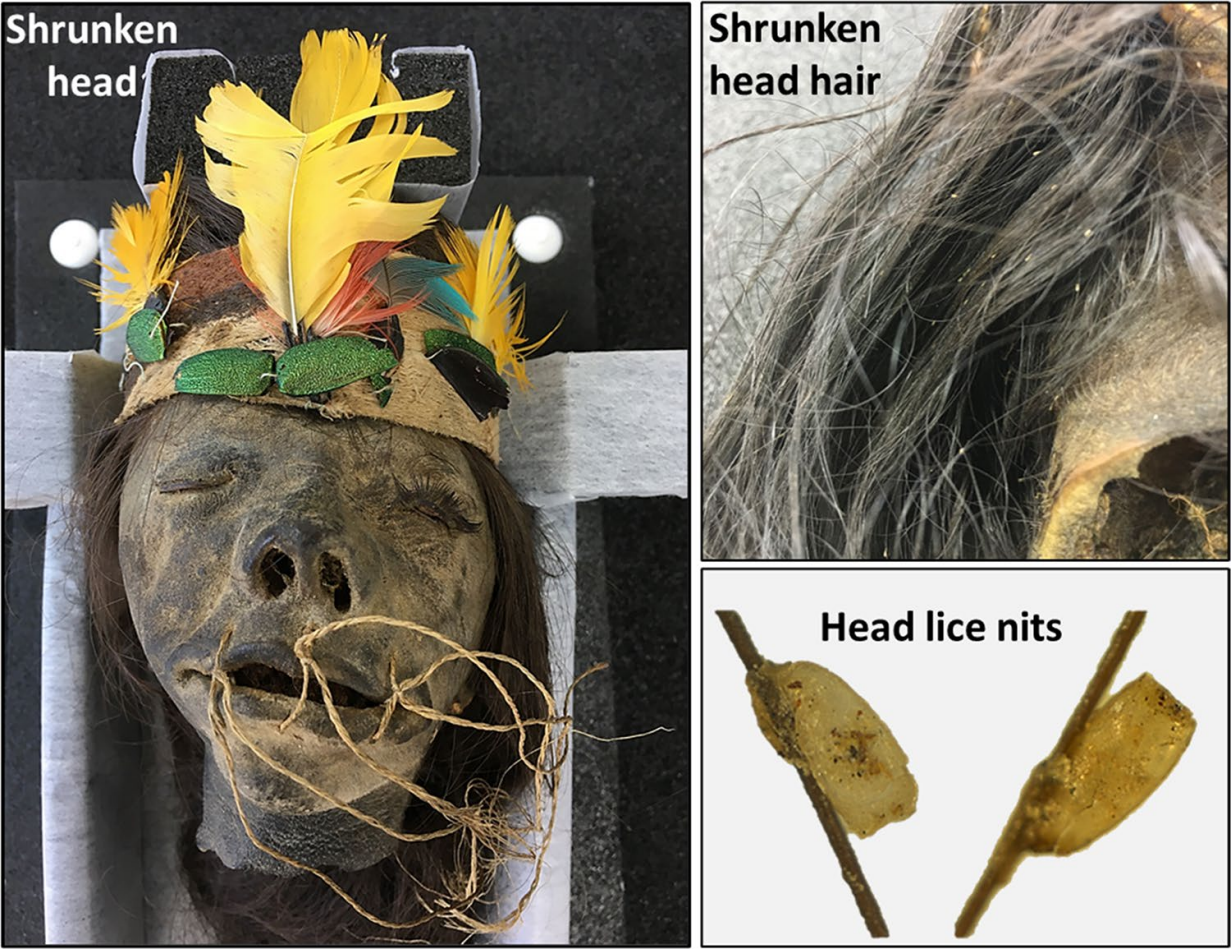
Shrunken head infested with head lice nits.

The mitochondrial DNA (mtDNA) analysis, 17 head lice nit samples collected from 6 mono and/or double infested human shrunken heads (Fig. 1), showed that nine of the ancient louse nits (52.94%) belonged to clade A, two (11.76%) belonged to clade B and six (35.29%) belonged to clade F (Table 1). Among the 6 human shrunken heads, three were mono-infested by only one clade of lice, two showed dual infestation with both clades and one were simultaneously infested with all the three clades (Table 1).

**Table 1:** Summary of clades identified in head lice nits from infested shrunken heads.

Shrunken head code	Geographical and ethnic origin; age	Louse number (lab code)	Clade identified	Haplotype identified
71.1932.108.172 D	Ecuador; Shuar; 19–twentieth century	TRL1	F	**F8 (new)**
		TRL2	F	F54
71.1938.101.1	Ecuador; Shuar; twentieth century	TRL3	A	**A84 (new)**
		TRL4	A	**A84 (new)**
		TRL5	A	**A80 (new)**
70.2003.11.1	Ecuador; Shuar; prior to 1946	TRL6	B	**B41 (new)**
		TRL7	A	**A82 (new)**
71.1946.52.2	Ecuador; Shuar; prior to 1946	TRL8	A	**A81 (new)**
		TRL9	A	**A81 (new)**
		TRL10	A	**A83 (new)**
71.1946.52.1	Ecuador; Shuar; prior to 1946	TRL11	F	F54
		TRL12	A	A5
		TRL13	F	F54
71.1950.0.398 X	Ecuador; Shuar; prior to 1950	TRL14	A	**A79 (new)**
		TRL15	F	F54
		TRL16	F	F54
		TRL17	B	**B42 (new)**
***Total***		**17**	**6 (F), 9 (A), 2 (B)**	

**Figure 2:**
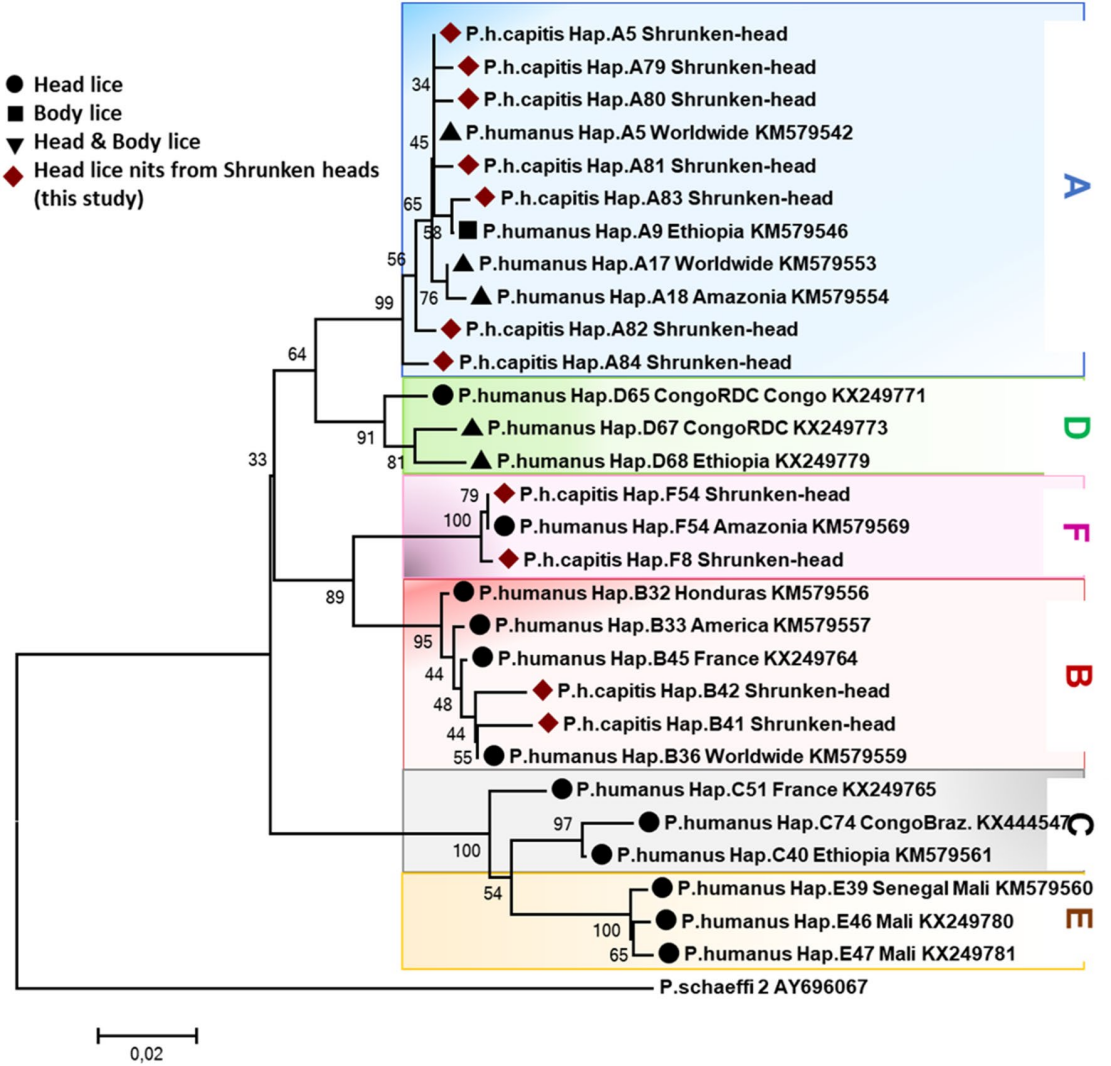
Phylogenetic analysis of head lice nits from infested shrunken heads. Phylogenetic tree showing the relationship between haplotypes identified in this study with other *Pediculus humanus* haplotypes. Phylogenetic inference was conducted in MEGA7 using the maximum likelihood method under the HKY + G model with 500 bootstrap replicates. The scale bar represents a 2% nucleotide sequence divergence. Haplotypes found in this study are marked by a dark red diamond.

An in-depth analysis of all obtained nucleotide sequences and their alignment with all publicly available haplotypes revealed the presence of eleven haplotypes from 17 samples, defined by the variation of 37 nucleotide positions, indicating an unusually high genetic diversity of the population of ancient head lice nits studied. Nine of these haplotypes were novel and unique to the ancient nits examined in this study, referred here to as A79-A84, B41, B42 and F8. The remaining two haplotypes possessed the widespread haplotype A5 of Clade A and haplotype F54 of Clade F, which is the most prevalent clade F haplotype (Table 1). These haplotypes, together with references from all the body and head lice haplogroups were used to construct a maximum-likelihood (ML) tree (Fig. 2).

## Discussion

To the best of our knowledge, this is the first report of molecular data from ancient human head lice nits recovered from shrunken heads of Shuar/Jivaro individuals from between the end of the nineteenth century and the middle of the twentieth century (Fig. 1). These are indigenous Amazonian tribes living in the headwaters of the Marañon River and its tributaries, in the mountainous region of northern Peru and eastern Ecuador^36^.

The mtDNA analysis revealed that the 17 ancient louse nits from shrunken human heads investigated in this work belong to three different cytb clades, A, B and F, distributed through 11 haplotypes, of which thirteen were novel haplotypes. When compared to another contemporary Amerindian population from the Wayampi community in Trois-Sauts village (French Guiana) reported by us in a previous study, where only clades A and F were identified, this sample shows in addition the presence of clade B in Amerindian populations^19^. While in another study of Pre-Colombian head lice from Peruvian mummies only clades A and B were identified^33^. No sequences from clades C, D and E were found, thus these clades remain restricted to the other continents, corroborating findings reported elsewhere^1,9,10,19,20^.

Haplogroup A was the most prevalent (52.94%), and yielded seven haplotypes, in which six haplotypes (provisionally called A79-A84 in this paper) were unique to the ancient head lice examined in this study, while haplotype A5, is the most prevalent and well distributed worldwide^19^. This haplotype was also present in ancient head lice from Peruvian mummies from the American continent^33,35^. In clade B sequences, two novel haplotypes were identified, referred to here as B41 and B42. Previous studies showed that this clade is prevalent and highly diversified among the contemporary head lice from the American continent^19,22^, a result which is supported by its detection among the ancient head nits we analysed here. Moreover, this clade was also present in pre-Columbian head lice from Peruvian mummies, which lead researchers initially to infer an American origin for this clade^33^. However, its discovery among the remains of head lice nits found in Israel and dating back 2,000 years, suggesting a Middle Eastern origin for this clade, followed by its spread to the American continent by the first humans, who had reached this continent through the Bering straits thousands of years ago^35^.

Finally, this study reveals the presence of a mitochondrial genotype clade F. The sequence analysis yielded two haplotypes, one of which was novel, referred to here as F8, while another had an F54 haplotype, which is the most prevalent clade F haplotype^19^. Interestingly, this clade is so far only found in south America, where it was recovered from only a few head lice sequences in Mexico and Argentina, while it was the most dominant lineage in lice collected from an isolated native Amazonian population from the Wayampi community in Trois-Sauts, French Guiana, where the F54 haplotype was the most prevalent^19^. This clade was also shared with *Pediculus mjobergi*, a New World monkeys’ louse, which is thought to be transmitted to monkeys from the first humans that had reached the American continent thousands of years ago^19^. In this study, head lice nits dating from between the nineteenth and twentieth centuries were collected from Shuar individuals belonging to an isolated Amerindian group. It is also well known that Amazonia is one of the few places in the world that has not been heavily affected by globalisation. Gives these facts, the most predominant hypothesis is that clade F may represent Native South America louse mitochondrial diversity. Moreover, as this clade is the sister group of clade B, it has been argued that it may be derived from clade B which was carried to America by an ancestral Eurasian Beringian population thousands of years ago^19^.

## Conclusion

Our finding confirms the presence of clades A, B and F in native South American populations. The description of clade F in our ancient head lice nits, together with previous reports from another Amerindian population from French Guiana, strongly support the hypothesis of a Native South America origin for this clade. Further support to our conclusion and new insights might come from the analysis of a large collection of modern and ancient native American lice.

## Materials and method

### Louse samples

In 2018, during a forensic and anthropological screening, a total of 17 ancient head lice nits were recovered from six shrunken human heads (known as *tsantsas*), preserved in the collection of the Musée du Quai Branly—Jacques Chirac (Paris, France)^37^. No adults or nymph stages of the head louse were observed (Fig. 1). These shrunken heads are from Shuar/Jivaro individuals living in the Amazon region of Ecuador, and date back to between the end of the nineteenth century and the middle of the twentieth century.

### DNA extraction

To ensure the accuracy of the results, all precautions were taken to prevent contamination by modern louse DNA. Each experimental procedure was performed in a separate, clean room, free of *P. humanus* and its DNA using laminar-flow hoods, using autoclaved and UV treated material. Each louse sample was washed twice in 99.8% ethanol for 10 min, rinsed three times with distilled water and dried on sterile filter paper, and then crushed individually in sterile Eppendorf tubes. A pre-lysis of louse sample was performed in 200 μl of buffer G2 and 10 μl Proteinase K supplied in the Qiagen DNeasy tissue kit (Qiagen, Courtaboeuf, France). DNA extraction was automatically performed in the EZ1 apparatus (Qiagen, Courtaboeuf, France) using the DNeasy tissue extraction kit according to the manufacturer’s instructions. The DNA was eluted in 100 μl of in water and stored at 4 °C until use for PCR amplifications. In order to detect possible contamination by external DNA, extractions and PCR amplification blanks were used as negative controls throughout the whole process.

### Lice clade and phylogenetic analysis

To identify the mitochondrial clades and haplotypes, all the DNA samples were subjected to standard PCR and sequencing targeting a 347-bp fragment of the *cytb* gene, using the primers described previously^38^. PCR reaction consisted of 50 µl volume including 25 µl Amplitaq gold master mix (Applied Biosystems, Foster City, CA, USA), 0.5 μM of each primer, 5 μl of DNA template, and water. The thermal cycling profile was one incubation step at 95 °C for 15 min, 40 cycles of one minute at 95 °C, 30 s at 56 °C and one minute at 72 °C followed by a final extension for five minutes at 72 °C. All PCRs were performed in a MiniAmp™ Plus Thermal Cycler (Thermo Fisher Scientific, Illkirch, France). Negative and positive controls were included in each assay. The success of PCR amplification was then verified by electrophoresis of the PCR product on a 1.5% agarose gels, stained with SYBR Safe (Invitrogen, San Diego, CA, USA) and visualized under transilluminator UV light. All PCR products were purified using the PCR filter plate Millipore NucleoFast 96 PCR kit (Macherey–Nagel EURL, Hoerdt, France) following the manufacturer’s recommendations. The amplicons were sequenced using the Big Dye Terminator Cycle Sequencing Kit (Perkin Elmer Applied Biosystems, Foster City, CA) with an ABI Prism 3130xl Genetic Analyzer capillary sequencer (Applied Biosystems) as per the manufacturer’s instructions. The obtained electropherograms were assembled and corrected using ChromasPro software (ChromasPro 1.7, Technelysium Pty Ltd., Tewantin, Australia) and compared with those available in the GenBank database by NCBI BLAST (http://blast.ncbi.nlm.nih.gov/Blast.cgi). For all *cytb* nucleotides sequences obtained in this study, unique haplotypes were defined using DnaSP v5.10 software^39^. All the identified haplotypes, together with the references from all the body and head lice clades^19^ were used to construct maximum-likelihood (ML) tree. To generate the best ML tree, the Modeltest v.3.7^40^ was used to examine model of nucleotide substitution and choose a best-fit model of sequence evolution. Tree reconstruction was conducted using MEGA 7 software (https://www.megasoftware.net) with ML method under HKY + G model with 500 bootstrap replicates. *Cytb* sequence from *P. schaeffi* (accession number: AY696067) was included as outgroup. Sequences of novel haplotypes are available in GenBank (Gen Bank accession MZ004849-MZ004859).

### Ethics statement

The lice samples were taken from museum objects with the authorization of the conservation, during a campaign of restoration and systematic study of the collections. Authorization of examination and sampling of the museum artifacts is given by one of the co-authors (P C), who is the director of the museum's research department. The *tsantsas* (human shrunken heads) have been collected prior to the 60's and are entered in the French national collections from anthropological fields and private collectors and were acquired under regular conditions according to up-to-date ethical code given by the ICOM (International Council of Museum) regarding human remains. No ethical issues were raised by this study, as no human remains were sampled or analysed, only the parasites adhering to the surface of the hair of these museum objects^41^.
